# Surface Attachment of Gold Nanoparticles Guided by Block Copolymer Micellar Films and Its Application in Silicon Etching

**DOI:** 10.3390/ma8073793

**Published:** 2015-06-24

**Authors:** Mingjie Wei, Yong Wang

**Affiliations:** State Key Laboratory of Materials-Oriented Chemical Engineering and College of Chemical Engineering, Nanjing Tech University, Nanjing 210009, Jiangsu, China; E-Mail: mj.wei@njtech.edu.cn

**Keywords:** metallic nanoparticle, block copolymer, surface patterning, silicon etching, micelle

## Abstract

Patterning metallic nanoparticles on substrate surfaces is important in a number of applications. However, it remains challenging to fabricate such patterned nanoparticles with easily controlled structural parameters, including particle sizes and densities, from simple methods. We report on a new route to directly pattern pre-formed gold nanoparticles with different diameters on block copolymer micellar monolayers coated on silicon substrates. Due to the synergetic effect of complexation and electrostatic interactions between the micellar cores and the gold particles, incubating the copolymer-coated silicon in a gold nanoparticles suspension leads to a monolayer of gold particles attached on the coated silicon. The intermediate micellar film was then removed using oxygen plasma treatment, allowing the direct contact of the gold particles with the Si substrate. We further demonstrate that the gold nanoparticles can serve as catalysts for the localized etching of the silicon substrate, resulting in nanoporous Si with a top layer of straight pores.

## 1. Introduction

Metal nanoparticles, such as Pt, Au, Ag, Fe, Co nanoparticles have found extensive applications in the fields of catalysis [1], magnetic data storage [2], sensing [3], optoelectronics [4], DNA detection [5], *etc*. In the catalyzed growth of nanostructure arrays [6], and in metal nanoparticle waveguides [7], it is highly desirable for the metal nanoparticles to be laterally distributed on flat substrate surfaces. Various routes, including physical deposition from elemental metals and chemical reduction from precursors of metal salts, have been developed to deposit nanoparticles onto substrate surfaces [8,9]. Among them, block copolymer (BCP)-assisted deposition of metal nanoparticles is a relatively simple but effective approach [10,11,12,13,14]. Usually amphiphilic diblock copolymers which contain both a hydrophobic and a hydrophilic block are used [15,16]. In a nonpolar solvent which selectively dissolves the hydrophobic blocks of the BCP, reverse micelles will form with the collapsed hydrophilic blocks as the core surrounded by an extended corona of the hydrophobic blocks. The size of the polar cores is determined by the length of the corresponding blocks, and normally in the range of 5 to 100 nm. These micelles can be transferred onto the surface of various substrates by a simple spin-coating or dip-coating process, producing a monolayer of densely packed micelles even after the complete evaporation of the solvent [12,13,17,18,19,20,21,22].

As the nanoparticles patterned on substrate with the assistance of BCP micellar templates can be effectively used as catalysts in the growth of well-aligned Si nanowires [23], carbon nanotubes [24,25] or zinc oxide arrays [26], much effort has been made in developing the BCP-guided patterning of metal nanoparticles, which can be roughly categorized into three further approaches. In the first two approaches, the metal precursors, usually metal salts, could either be loaded into the polar cores of BCP simultaneously with the formation of the micelles [17,18,19,21], or incubated into the BCP cores after the formation of BCP micelle by immersion of the film into the solution of the metal salt [12,13,20,22]. The metal precursor filled in the BCP micelles obtained through either of the two above described methods can then be converted to elemental metals by reduction, while the well-ordered pattern and the nanoscale size of the initial micelles are mostly kept. In the third method the BCP micelle films and metal nanoparticles are prepared separately, followed by immersion of the BCP film into the nanoparticle suspension. Provided there is a specific interaction between the micelle cores and the particles, e.g. an electrostatic attraction, the particles will selectively attach on the top of the micelle cores, even though the micelle cores might be wrapped by an inert corona of the other block [27,28,29]. This direct method should have several advantages over the two methods starting from metal precursors since the used particles are pre-formed. Pre-formed particles with different chemical compositions, sizes and even shapes in a relatively large range could be patterned on BCP films provided the particles have opposite charges to the hydrophilic domains which could be readily realized through surface modifications of the metallic nanoparticles. In contrast to both metal precursor-based methods, the size of the resulted nanoparticles is determined by the saturated amount of precursors loaded in or adsorbed in the micelle cores, which is limited to the size of the cores and can be tuned in a relatively narrow window. In addition, only limited numbers of precursors can be bound to the hydrophilic domain through complexation or electrostatic attraction. Furthermore, the resulting metal nanoparticles can only be obtained in spherical shape due to geometry of the sphere-like micelles. In the third method involving pre-formed nanoparticles, the BCP film between the nanoparticles and substrate can then be removed by plasma treatment, transferring the patterned nanoparticles to the substrate. The third method allows for the separate design and preparation of the particles and the BCP films, providing much higher flexibility in the patterning. However, as an emerging method, it is only applied in the fabrication of very limited types of patterns, predominantly stripe-like patterns with the purpose of producing metal nanowires [27,28], although patterns with other forms are desired in various applications. More importantly, there is a lack of kinetic understanding on the immobilization process of the metal particles on the BCP thin films which is very important in the practical applications of this method.

In this work, we expanded this method to fabricate isolated dot-like patterns and made a systematic investigation on the immobilization of gold nanoparticles with different diameters on the amphiphilic BCP micellar films including the kinetics of the immobilization of gold nanoparticles on the BCP films, the influence of the particle sizes and the temperature on the numbers of arrested particles on the BCP films. We first produced a monolayer of hexagonally patterned polystyrene-*b*-poly(2-vinyl pyridine) (PS-b-P2VP, denoted as S2VP hereafter) micelles on a silicon substrate by spin coating, then incubated the S2VP-coated silicon in a gold nanoparticle suspension, resulting in a monolayer of gold particles attached on the coated silicon. The intermediate S2VP film was then removed using oxygen plasma treatment, allowing direct contact of the gold particles with the Si substrate. The gold nanoparticle-deposited Si was finally etched in a mixture of HF and H_2_O_2_ to fabricate nanoporous Si where gold nanoparticles served as catalysts for the localized etching.

## 2. Results and Discussion

In *o*-xylene, which is a selective solvent for the nonpolar PS blocks, the insoluble P2VP blocks condense together and form cores surrounded by PS shells that extend to the solvent and keeps the core-shell-structured reverse micelle system stable in the selective solvent. Upon spin coating, the S2VP reverse micelles were dispersed onto the surface of the Si substrate, and a monolayer of hexagonally packed S2VP micelles formed because of a delicate balance between attractive capillary forces between neighboring nanoparticles (within the liquid film) and repulsive forces due to electrostatic or steric interactions [21,30]. When the S2VP-coated samples are immersed in gold colloid suspended in water, at least three effects have to be considered. The first is the water-induced swelling of P2VP cores since the three kinds of gold colloid suspensions investigated in this work all have pH in the range of 5 to 6, at which 2VP cores will swell and expand their volume, and, as a result, the upper PS layer reduces its thickness or even ruptures upon a longer immersion. The second effect is also induced by water. The slightly acidic water may also protonize the pyridyl groups of P2VP, making them positively charged. The third is the attachment of the gold colloid to the S2VP film, especially to the position where the P2VP blocks locate. Since the gold colloids as received are negatively charged, they have an affinity towards the positively charged pyridyl groups on P2VP due to static attraction.

**Figure 1:**
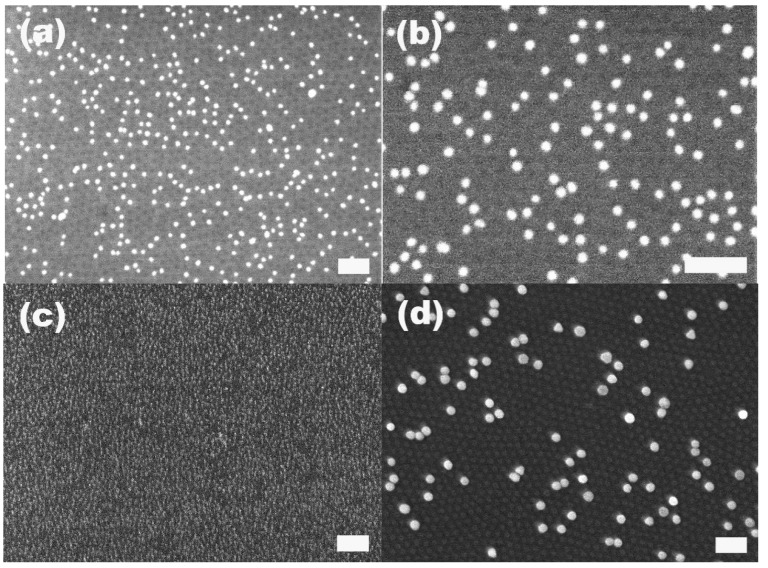
Attachment of gold particles on S2VP film after immersion for 2 h. The gold particle size is (**a**,**b**) 20 nm; (**c**) 10 nm; (**d**) 60 nm, respectively. The scale bar is 200 nm.

We immersed the S2VP-coated substrates into gold colloids with diameters of 10, 20 and 60 nm, respectively, and found that for the three kinds of gold colloids with different sizes the S2VP could effectively collect gold colloid from the bulk suspension and transfer them to the surface of the substrate. However, as demonstrated by Figure 1, it is more efficient for S2VP film to collect smaller gold particles, that is, the smaller the particle size, the more the amount of particles could be harvested by S2VP films with the same area within the same immersion time. As shown in Table 1, after 2 h immersion, the amount of the collected gold particles was 900, 100 and 18 particles per μm^2^ for 10, 20 and 60 nm gold particles, respectively. One reason for that is the higher original concentration of the smaller gold particles in the suspension; more particles result in more chances to be arrested by the S2VP film. Another reason is that once attachment happens, the smaller particles have less energy to detach compared to bigger ones. It is worth noting that even in the case of smaller gold particles, e.g., 10 or 20 nm, although the gold particles are densely packed on S2VP film, aggregation occurs rarely both laterally and vertically due to the static repulsion between particles [31], which means we also obtain a monolayer of gold particles. The closest distance between two gold particles is around 10 nm in the case of attached 20 nm gold particles.

**Table 1:** The number of attached gold particles with different particle sizes on S2VP film.

Gold particle size (nm)	Concentration (particles/mL)	Attached particles (particles/ μm^2^)
10 nm	5.7 × 10^12^	900
20 nm	7.0 × 10^11^	100
60 nm	2.6 × 10^10^	18

Under a close SEM examination, e.g., in Figure 1d, we found that there were hexagonally ordered cavities on the S2VP film. These cavities may be produced during the immersion of S2VP in gold particles suspended in water. Water which is the selective solvent for the P2VP component will swell the P2VP core in the S2VP film, and induce the cavitation of the P2VP cores by the reconfiguration of the deposited S2VP micelles. Recently, there were a few reports on the polar solvents, including acetic acid, ethanol and diluted HCl/HF-induced cavitation of S2VP and polystyrene-b-poly (4-vinyl pyridine) micelle films [22]. We believe the reason for all the cavitation resulting from exposure to different polar solvents is the same, that is, the swelling of the P2VP core towards good solvents and the only difference is the degree of the swelling. The cavitation of the P2VP is critical to the strong attachment of gold particles to S2VP film simply because the cavitation directly exposes the P2VP component to the surroundings, making 2VP easy to be protonized and positively charged, and also directly accessible for the negatively charged gold particles. Without the cavitation, the P2VP and gold particles interact with each other spaced by a neutral PS layer, which definitely reduces the Coulomb attraction.

**Figure 2:**
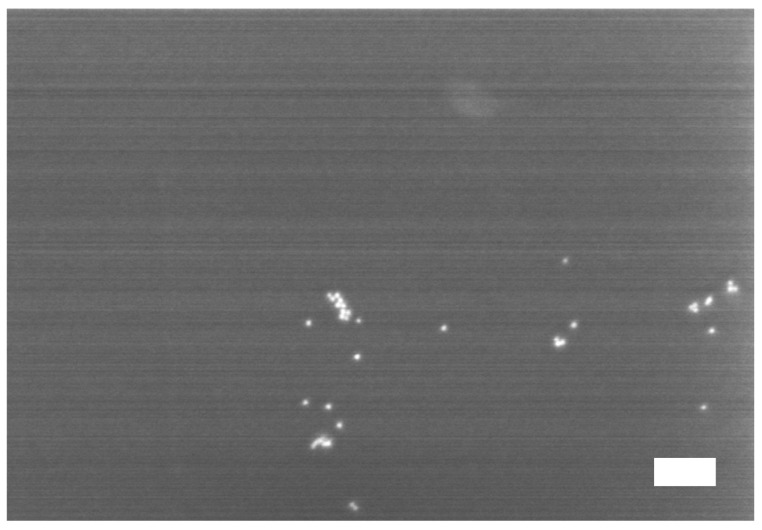
Attachment of gold particles on the native silicon surface without the S2VP coating layer. The native silicon was immersed in the solution of 10 nm gold nanoparticles for 2 h. The scale bar is 100 nm.

The S2VP micellar layer plays an essential role in the immobilization of gold nanoparticles on the silicon substrate. The native silicon surface without the coating of the S2VP layer exhibits very weak capability to immobilize gold particles. As shown in Figure 2, there are only very few gold particles present on the surface when the native silicon is immersed in the 10 nm gold solution for 2 h. Moreover, the arrested particles are mostly in the agglomerated state. This is in stark contrast with the case of S2VP-coated silicon in which gold nanoparticles are densely adsorbed on the surface without noticeable agglomerations (Figure 1c). The fact that gold nanoparticles cannot be directly patterned on the silicon surface is due to the static repulsion between the negatively charged gold particles and the hydroxyl groups terminated on the native silicon surface where an oxide layer (SiO_x_) is always present.

**Figure 3:**
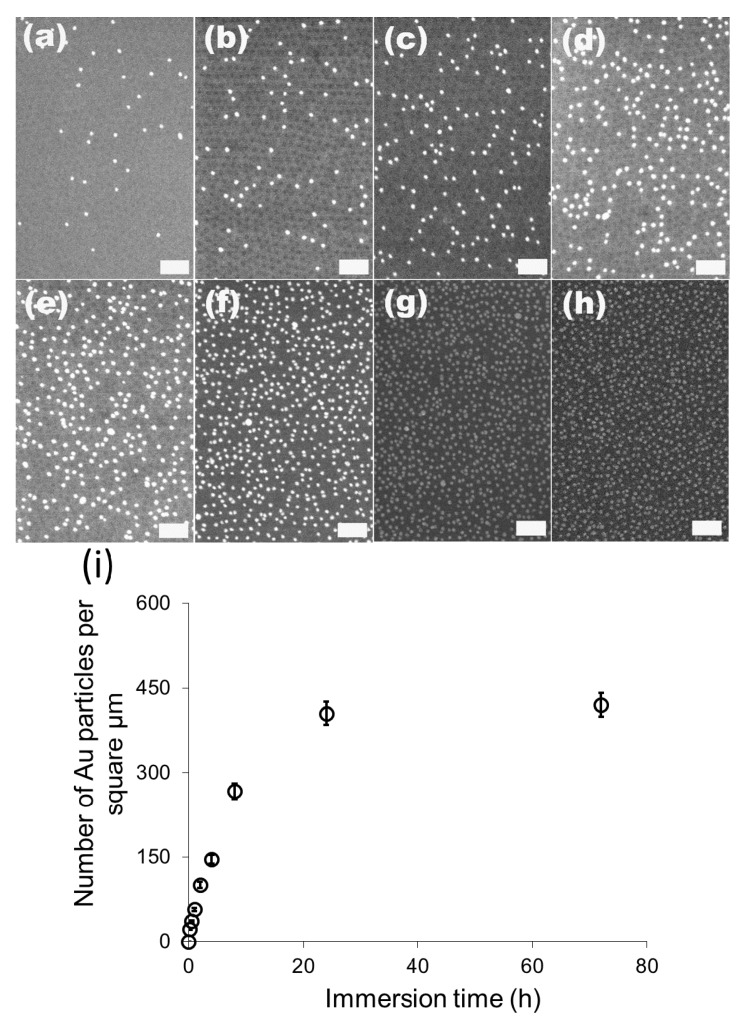
SEM images of 20 nm gold attached S2VP film after different immersion times (**a**–**g** correspond to 10 min, 0.5 h, 1 h, 2 h, 4 h, 8 h, 24 h and 72 h, respectively, and the scale bar is 200 nm); and the dependence curve of number of the attached gold particles with immersion time (**h**).

We then investigated the change of the number of the attached gold particles with the immersion time using 20 nm particles as the model system. Figure 3a–g show the SEM images of the 20 nm gold particles attached on S2VP film with immersion times from 10 min up to 72 h, respectively; and Figure 3h is the plot summarizing the dependence of the number of the attached particles with immersion time. The number of the attached particles increased quickly in the initial 8 h, from 20 particles per μm^2^ after 10 min immersion to 270 particles per μm^2^ for 8 h immersion, and further increased to around 410 particles per μm^2^ but with a lower increasing rate for 24 h immersion, where it reached the equilibrium; there was no obvious rise from extending the immersion time to 72 h. Before the equilibrium is achieved, there are still some “active sites” on the S2VP surface which can hold gold particles, and more “active sites” were occupied by gold particles with longer immersion time. When all the sites were used up, no more particles could be arrested and equilibrium was reached. Note that even the equilibrium was reached with a considerable amount of gold particles densely packed on the S2VP surface (see Figure 2f,h); aggregation of the particles still rarely happened and a particle monolayer was kept and became more even, meaning that gold particles preferred to stay on the bare surface of the S2VP rather than contact other particles that were previously attached to the S2VP surface [32]. We note that the ordering of the gold nanoparticles does not fully record the patterning of the underneath polymer micellar film as the noncovalent interactions between the gold particles and the polymer template is relatively weak and moderate disturbances in the operation may deteriorate the ordering of the gold particles.

**Figure 4:**
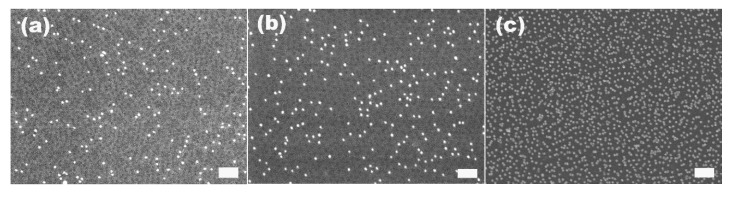
SEM images of S2VP film immersed in 20 nm gold particle suspension at 6 °C (**a**); 25 °C (**b**); 60 °C (**c**) for 1 h. The scale bar is 200 nm.

We found the temperature also greatly influenced the kinetics of the attachment of gold particles on S2VP. Figure 4a–c give the SEM images of S2VP film immersed in 20 nm gold particles for 1 h at 6, 25 and 60 °C, respectively. Generally, the higher the immersion temperature, the more the particles attached on a given area of the S2VP film within a certain immersion time. Specifically, in the case of immersion in 20 nm gold suspension for 1 h at 6, 25 and 60 °C, 12, 57 and 93 particles were attached to S2VP with an area of 1 μm^2^, respectively. At higher temperatures, the Brownian motion and diffusivity of the gold particles is enhanced; as a result, more particles will have the chance to approach or contact the 2VP domains in unit time, that is, more particles will be adsorbed by 2VP. However, as the static attraction between 2VP and gold particles is stronger than the Brownian motion [33], once adsorbed on S2VP, the gold particles lose their capability to escape, that is, they cannot desorb to return to the bulk of the solution even at elevated temperature. Therefore, more particles are arrested by the S2VP film at a higher temperature before the equilibrium is reached.

**Figure 5:**
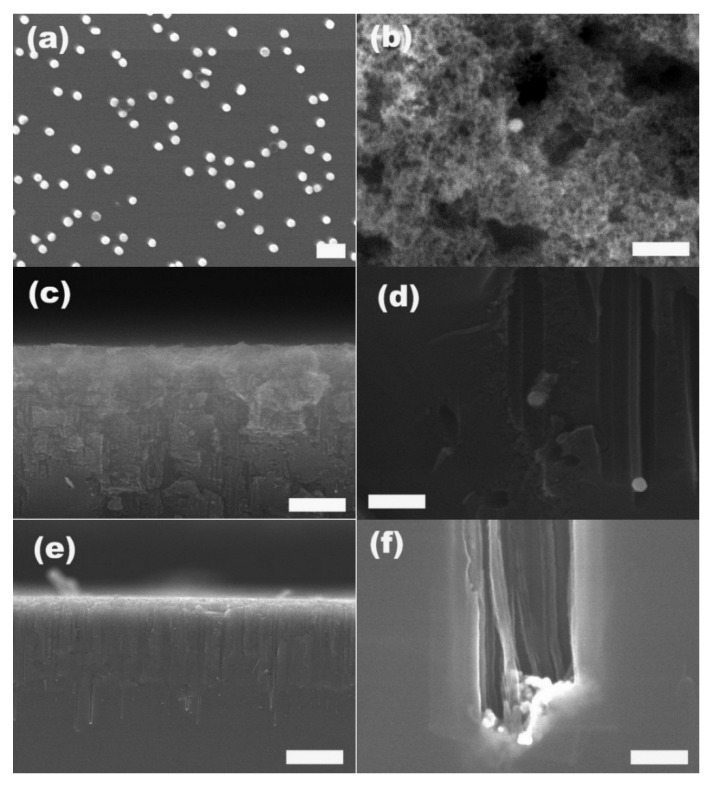
SEM images of S2VP-coated Si immersed in 60 nm gold suspension for 2 h after oxygen plasma etching (**a**), and after etching with HF/H_2_O_2_ for 30 mins; (**b**) surface; (**c**,**d**) cross-section at different magnifications; (**e**,**f**) cross-section of Si etched with HF/H_2_O_2_ in the presence of S2VP directed deposition of 20 nm gold nanoparticles. The scale bar in (**a**–**f**) is 200 nm, in (**c**) 1 μm, and in (**e**) 10 μm, respectively.

It was reported that noble metal nanoparticles, mainly Ag and Pt and rarely Au [34], deposited on the Si surface could catalyze the etching of Si, in which porous silicon with deep and straight nanopores were formed [35]. Considering that the method described above is an effective and more controllable way to deposit gold nanoparticles with pre-determined size on silicon surface, we investigated the application of the BCP-patterned preformed gold particles as catalysts in the etching of silicon to fabricate porous silicon. We first removed the S2VP film between gold particles and silicon substrate using an oxygen plasma treatment, allowing gold particles to come in direct contact with the silicon substrate surface. As already shown in Figure 1d, on the S2VP-coated Si surface which was immersed in 60 nm gold suspension for 2 h, both the attached gold particles and underneath S2VP film indicating by the hexagonally ordered cavities are visible, while in the plasma-treated sample as shown in Figure 5a, only gold particles are still present and the ordered cavities disappear, suggesting the effective removal of the polymer film by the plasma treatment. Furthermore, the specific pattern of the gold particles, including particle density and interparticle distance, survived from the harsh plasma treatment, that is, plasma treatment did not induce the aggregation of gold particles. After incubation in the etching solution, which was a mixture of H_2_O_2_ and HF for 30 min, the plasma-treated Si changed color to grayish yellow on the surface where the gold particles attached while the opposite surface remained unchanged. We found that the etched surface was completely porous with pore sizes down to a few nanometers to several tens of nanometers, and most of the gold particles initially attached on this surface were absent, as shown in Figure 5b. We then examined the cross-sectional morphology of the etched silicon. As revealed by Figure 5c,d, the etched Si had a first amorphous porous layer with a thickness of around 3 μm, under which there were straight channel-like pores perpendicular to the Si surface and parallel to each other, extending to a depth around 12 μm. At the end of almost each pore, there is a gold particle present. As shown in an enlarged SEM photograph of pore ends (Figure 5d), the pore diameter is around 60 nm, comparable to the size of the gold particles localized at the pore ends. Etching with 20 nm gold particles transferred to the Si surface by removing the S2VP film gave similar results and again the size of the etched poles, determined by the size of the gold particles, was around 20 nm. Due to the high density of the gold particles and short interparticle distance on the Si surface, some neighboring pores coalesced together and were present as larger pores with diameters around 200 nanometers, as shown in Figure 5f. Note that 20 nm gold particles resulted in about 20 μm deep pores in 30 min etching which is deeper than that of etching with 60 nm gold particles in the same etching period, and this may be attributed to the higher catalysis efficiency for smaller nanoparticles in the metal-assisted Si etching.

As we demonstrated in the beginning of this work, gold nanoparticles with different sizes could be easily attached to S2VP films coated on the Si substrate because the density of the attached particles and interparticle distance could be controlled in a relatively broad range, the structure parameters of the etched pores in silicon, e.g., size, density, depth and distance between each other, could easily be correspondingly tuned.

**Figure 6:**
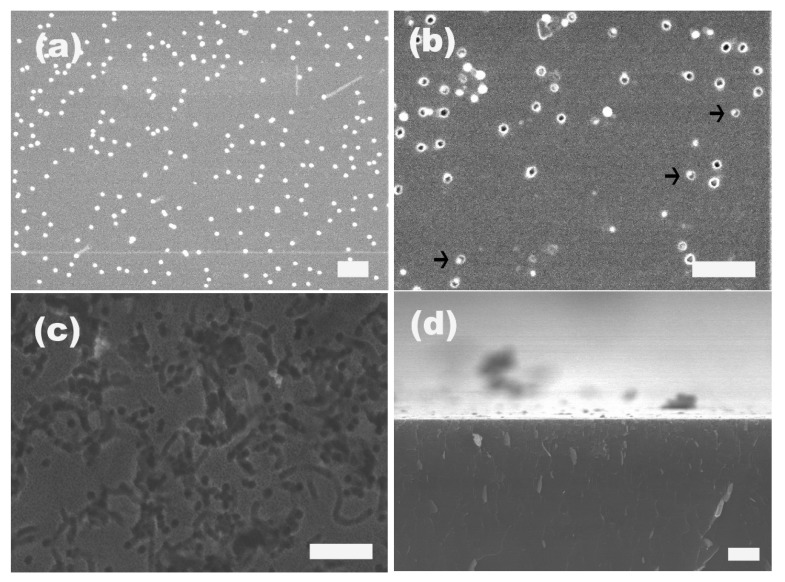
SEM images of Si surface after etching in the presence of 20 nm gold particles for (**a**) 30 s; (**b**) 60 s; (**c**) 5 min; and (**d**) is the cross-sectional SEM image of (**c**). The scale bar in (**a**–**c**) is 200 nm, in (**d**) 1 μm.

Furthermore, we tracked the etching process by examining the Si substrates at different etching times. Figure 6a–c give the SEM images of Si substrates etched in the presence of 20 nm gold particles, for 30 s, 60 s and 5 min, respectively. When the gold-attached Si was exposed, the etching solution for a very short time, e.g., 30 s, the surface morphology of the Si surface still looked similar to that of the gold-attached Si after the plasma treatment, and no noticeable changes could be observed under SEM (Figure 6a). In the first 30 s or so, the main reaction taking place in the etching solution should be the dissolution of the oxide layer on top of the Si surface by HF. Even though the gold-assisted oxidization of exposed Si by H_2_O_2_ underneath the gold particles occurred in the initial 30 s immersion into etching solution, it should be to a very limited extent since there was no evident sinking of the gold particles into the substrate. If the immersion time extended to 60 s, many holes with diameters similar to the size of gold particles appeared on the Si surface, and in some of the holes, there was one particle inside (indicated by arrows), as shown in Figure 6b. These surface holes suggested that in 60 s etching, oxidization of Si took place underneath each gold particle, and the resultant silicon oxide was removed by HF simultaneously, leading to the sinking of gold particles and formation of holes. However, since the etching time was still too short, the resulted holes were not deep and their depth should be comparable to the diameter of the gold particles here, in the magnitude of 20 nm, since in many of the holes, one gold particle was still visible under the detection of SEM. On the other hand, since there were some variations of the particle size of gold, and as we discussed above, smaller gold particles lead to faster etching rate, the gold particles were only observable in some of the holes, and for the other pores, the depth was larger and the gold particles which always stayed at the bottom of the pores were invisible. When the etching time was extended to 5 min, gold particles kept sinking, which led to deeper holes and all of them submerged into the Si substrate. As a result, no gold particles could be found on the surface and only the openings of the holes were visible. In addition, besides those hole openings, the whole Si surface was covered by pores with a diameter of a few nanometers, as indicated in Figure 6c. From its sectional SEM image (Figure 6d), it could be determined that the hole depth after 5 min etching was approximately 1 μm. Considering our previous result, etching for 30 min resulted in pores with a depth of around 20 μm, we recognized that the etching did not process in a linear way, and the etching rate might be accelerated with the prolonged etching time. On the other hand, it is worth noting the color of the Si surfaces changed with different etching time. For short etching, e.g., 30 and 60 s, the Si substrates kept their color like the initial Si surface without any treatment, and after etching for 5 min, the Si surface took a dark blue to black color while the color turned to grayish yellow after etching for 30 min. This color change with the extension of etching time might have resulted from the different surface morphologies obtained with different etching times. However, this metal-assisted etching method is currently not able to produce straight pores with large aspect ratios into the deep interior of the silicon substrate [36,37]. However, the patterns of the gold particles can be at least maintained on the near surface region of the silicon substrate, which is also very interesting in many applications where pore ordering is not essential, like photovoltaics.

## 3. Experimental Section

Gold colloid suspension with particle sizes of 10 nm, 20 nm and 60 nm were obtained from International BBI with a concentration of 5.7 × 10^12^, 7.0 × 10^11^, and 2.6 × 10^10^ particles/mL, respectively. These particles are synthesized by the reduction of HAuCl_4_ with citric acid and their surface is covered with citrate ligands and consequently they are negatively charged. The block copolymer, PS (*M*_n_ = 102,000)-*b*-P2VP (*M*_n_ = 97,000) with polydispersity *M*_w_/*M*_n_ = 1.12 was purchased from Polymer Source, Inc., and used without purification. The S2VP was dissolved in *o*-xylene to obtain a 0.5 wt%/wt% solution, and then spin coated on silicon (100) wafer dices (about 1 cm × 1 cm size ) at 2000 rpm. Before spin coating, the Si dices were cleaned in ethanol and dried by nitrogen blowing. The coated Si dices were then immersed in a gold colloid suspension without any agitation with a certain particle size kept at a desired temperature for given time periods, followed by a gentle rinse in deionized water. In a control experiment, the same immersion (20 nm gold nanoparticles) for 2 h and rinse treatment was also performed on clean Si dices without coating the BCP film. The Si dices were subsequently treated with oxygen plasma with 0.7 Torr oxygen pressure and 100 W output power for 10 min to remove the polymer layer between the attached gold particles and the Si substrate. For the etching of Si, the plasma-treated samples were kept into a mixed solution of 10% HF and 30% H_2_O_2_ (10:1, v/v) at room temperature for 30 min. The morphologies of the samples at different stages were examined on a JSM6340F scanning electron microscope operated at 15 keV accelerating voltage.

## 4. Conclusions

In summary, negatively charged gold nanoparticles with different sizes (10, 20 and 60 nm) could be effectively attached on the polystyrene-*b*-poly(2-vinyl pyridine) (S2VP) film spin coated on silicon substrate by incubation in gold particle suspension. The number of the attached gold particles increased with incubation time initially and finally leveled off, and higher incubation temperature will also accelerate the attachment of gold particles on S2VP. After removal of the S2VP film by oxygen plasma treatment, the gold particles kept their initial arrangement on Si surface and no aggregation occurred. Etching the gold nanoparticle-patterned Si in HF/H_2_O_2_, nanoporous Si with straight pore perpendicular to Si surface, capped with an amorphous porous layer, was achieved with one gold nanoparticle staying at the end of each pore. The diameter of the pore was in accordance with the size of gold nanoparticles, and the interpore distance was determined by the distance between initial gold nanoparticles. Smaller gold nanoparticles catalyzed to form deeper pores than bigger ones, indicating smaller gold nanoparticles were more effective in the catalysis of Si etching.
